# Prevalence of asymptomatic Clostridium difficile colonization in tertiary hospital patients in Australia

**DOI:** 10.1186/2047-2994-4-S1-O33

**Published:** 2015-06-16

**Authors:** L Furuya Kanamori, DL Paterson, TV Riley, NF Foster, C Huber, J Marquess, T Harris-Brown, S Havers, SJ McKenzie, L Yakob, AC Clements

**Affiliations:** 1 Research School of Population Health, The Australian National University, Canberra, Australia; 2 University of Queensland Centre for Clinical Research, University of Queensland, Herston, Australia; 3 School of Pathology and Laboratory Medicine, University of Western Australia, Perth, Australia; 4 Centre for Healthcare Related Infection Surveillance and Prevention, Queensland Health, Queensland, Australia; 5 School of Population Health, University of Queensland, Herston, Australia; 6 Department of Disease Control, London School of Hygiene and Tropical Medicine, London, UK

## Introduction

Despite the importance of *C. difficile* infection (CDI) as a cause of hospital-acquired diarrhea, few studies have investigated the prevalence of asymptomatic *C. difficile* colonization in a broad cross-section of the general hospital patient population over multiple years and seasons.

## Objectives

To estimate the prevalence of asymptomatic *C. difficile* colonization of tertiary hospitals in different Australian States during six time-periods (late summer [Feb-Mar] and late winter [Aug-Sep]) 2012-2014 and to describe the diversity of PCR ribotypes isolated from asymptomatic patients.

## Methods

A three-year repeated cross-sectional study with biannual surveys of randomly selected adult patients from all care wards in tertiary hospitals in Australia was conducted. Stool specimens were cultured for *C. difficile* and isolates were characterized by PCR ribotyping. Overall prevalence of asymptomatic *C. difficile* colonization, hospital and time-period specific prevalences were calculated and compared using logistic regression.

## Results

Asymptomatic *C. difficile* colonization was identified in 112/1417 (7.90%; 95% CI 6.55–9.43) patients during the study period. Asymptomatic *C. difficile* colonization prevalence was at its highest in Feb-Mar 2012 (11.95%; 95% CI 8.46-16.22), whereas the lowest prevalence was observed in Aug-Sep 2014 (5.84%; 95% CI 3.30-9.44). A seasonal pattern characterized by lower prevalence in late winter (OR 0.63; 95% CI 0.42–0.94) was identified. The majority of the isolates (77.55%) were toxigenic *C. difficile* strains, PCR 014 and 018 were the most frequent toxigenic strains isolated.

## Conclusion

High variability of asymptomatic *C. difficile* colonization prevalence was observed across seasons. The majority of the asymptomatic *C. difficile* infected patients were colonized by toxigenic strains.

*The study was funded by a NHRMC project grant (APP1006243).

## Disclosure of interest

None declared.

